# A national survey of household pet lemur ownership in Madagascar

**DOI:** 10.1371/journal.pone.0216593

**Published:** 2019-05-08

**Authors:** Kim E. Reuter, Marni LaFleur, Tara A. Clarke, Fabiola Holiniaina Kjeldgaard, Irène Ramanantenasoa, Tiana Ratolojanahary, Jonah Ratsimbazafy, Lucia Rodriguez, Toby Schaeffer, Melissa S. Schaefer

**Affiliations:** 1 Temple University, Philadelphia, Pennsylvania, United States of America; 2 Conservation International, Nairobi, Kenya; 3 The Biodiversity Consultancy, Cambridge, United Kingdom; 4 Pet Lemur Survey Initiative, housed by the University of Utah, Salt Lake City, Utah, United States of America; 5 University of San Diego, San Diego, California, United States of America; 6 Lemur Love, Inc., San Diego, California, United States of America; 7 Duke University, Durham, North Carolina, United States of America; 8 Association Mitsinjo, Madagascar; 9 Groupe d’étude et de recherche sur les primates de Madagascar, Madagascar; 10 Lemur Conservation Network, Washington, DC, United States of America; 11 University of Utah, Salt Lake City, Utah, United States of America; 12 Salt Lake City Community College, Salt Lake City, Utah, United States of America; Universitat Autònoma de Barcelona, SPAIN

## Abstract

Primates are extracted from the wild for the pet trade across the world. In Madagascar, lemurs are kept as illegal pets and an understanding of lemur pet ownership at the national level is lacking. In 2013 and 2016, we undertook a national survey in 11 of Madagascar’s 22 administrative regions (n = 28 towns) with 1,709 households. To our knowledge, this is the first national survey of the household ownership of pet primates in a country where they are endemic. In the 1.5 years prior to being surveyed, 8% ± 4% (towns as replicates) of respondents had seen a captive lemur while a further 0.7% ± 0.5% of respondents had owned one personally. We estimate that 33,428 ± 24,846 lemurs were kept in Malagasy households in the six months prior to our survey efforts, with 18,462 ± 12,963 of these pet lemurs estimated in urban household alone. Rates of lemur ownership did not differ by province but increased with the human population of a town and with the popularity of the town on Flickr (a proxy indicator for tourism). We found that the visibility of pet lemur ownership did not differ across the country, but it did increase with the size of the town and popularity with tourists. Areas with visible pet lemurs were not always the areas with the highest rates of pet lemur ownership, highlighting that many pet lemurs are hidden from the general public. Our study highlights the need for conservation programs to consider both the proportion of inhabitants that own pet lemurs and the total number of lemurs that are potentially being kept as pets in those towns. We close by noting that for some species, even just a small amount of localized live extraction for pet ownership could be enough to cause localized population extinctions over time. Moreover, an urgent response is needed to combat a recent and alarming rise in illegal exploitation of biodiversity across Madagascar.

## Introduction

Globally, primates are often prized as pets [1,2]. Live primates sourced for the pet trade are either bred in captivity or extracted from the wild. Over a decade ago, live capture was estimated to affect 40,000 primates/year globally [3], though this is likely now an underestimate [2]. When extracted from the wild (e.g. from range countries), primates can be traded domestically (within national borders) or exported to other countries for sale and trade [1]. These captures, trades, and sales may be legal or illegal, depending on the region, country, and status of the primate species (i.e. if they are listed by CITES) (see [4]). Relatively little is known about the trade of live primates–especially the trade occurring domestically within range countries–often because of difficulties collecting accurate data (e.g., [5, 6]) and the clandestine nature of activities that may be illegal (reviewed by [7]).

Madagascar’s primates–the lemurs–are described as one of the most threatened groups of large vertebrates in the world [8]. In Madagascar, it has been illegal to keep lemurs as pets since 1962 (Ordonnance no 1962–20), though they are kept as pets by a variety of owners. Lemurs are illegal to own, capture, sell, and trade (lemurs can be confiscated and owners can be fined, Ordonnance no 60–128 1962). Lemur ownership in Madagascar is still geographically widespread [4], with lemurs kept in captivity for a range of reasons, including as personal pets and for money-making [9]. Pet lemurs in Madagascar are rarely bred in captivity [9] and appear to be nearly exclusively sourced from the wild [5]; there is little legal or illegal export of live lemurs from Madagascar [10]. Between 2000–2017, 30 individual lemurs were exported from Madagascar for the purposes of breeding, medical use, scientific research, and display/use in zoological facilities [11].

Although only a minority of people own lemurs in Madagascar (estimated at 3% ± 2% of the general population [5]), the impact of this extraction is likely substantial. By extrapolating a sub-national dataset for Madagascar, it was estimated that roughly 28,000 lemurs were kept as illegal pets in urban areas of between 2010 and mid-2013 [5]. The same paper also hypothesized that ownership is more common in urban than in rural areas [5]. More recently, it has become evident that the illegal extraction of lemurs from the wild for captivity is disproportionately affecting some species [12–14]. *Lemur catta* is the species most commonly kept as a pet and population modeling suggests that relatively modest levels of extraction for pet ownership (i.e. 10% of infants/juveniles targeted in populations of 500 or less animals), will result in localized population declines for the species [12]. This is problematic in the context of the pet trade adding additional pressure to populations that are already facing hunting and habitat degradation threats [14]. Aside from *L*. *catta*, species commonly kept as pets include *Varecia variegata* and *Eulemur fulvus* [13]. It has been hypothesized that diurnal lemurs living in groups might be more likely to be captured as pets than other species [13]. Other lemur groups that are less commonly kept as pets, such as Indriidae and Lepilemuridae [13], are typically those that have very poor captive or translocation survivorship, as they are unable to transition changes in diet and/or the associated changes in gut microbiome (see [15,16]).

Despite the growing literature on the topic, a country-level assessment of the illegal ownership of pet lemurs is still needed. First, it is still unclear whether existing estimates [4] referenced above (the number of lemurs kept in urban, illegal captivity) are accurate, even as these are being used to frame conservation programming. These estimates were made from a sub-national survey effort focused in the northern part of the country and hence a national survey effort would provide valuable insights into the scale of the illegal pet lemur issue in the country. Second, it remains unclear whether there are areas of the country where a higher proportion of people own lemurs as pets, though one study suggested this could be the case [10]. This kind of information can help target limited conservation and enforcement resources, identify which species might be most at risk, and provide some insights into the drivers of pet lemur ownership. Finally, whether there are areas in the country where a higher proportion of people have seen pet lemurs has not been examined yet, even though areas where many people have seen lemurs (i.e. areas where pet lemurs are not concealed) may not be the same places in which rates of ownership are high. This might happen, for example, in areas where pet lemur ownership is primarily motivated by money-making from the tourism industry (e.g. lemurs have to be generally kept in a non-concealed place for tourists to appreciate them) though social status and fear of enforcement could also be factors [17].

In this study, we undertook a national survey of pet lemur ownership in Madagascar. The aim of this survey was to better estimate the number of lemurs kept as illegal pets in the recent past, while also aiming to expand our understanding of how the rates of ownership and visibility of pet lemur ownership might differ across the country. Specifically, we were interested in examining three different aspects of pet lemur ownership: 1) whether the rates of ownership differed across the country; 2) whether the proportion of people who had seen pet lemurs differed across the country; and 3) whether there were areas with a higher absolute number of lemur owners. In addition, based on the prior studies described above, we articulated several hypotheses regarding how the first two aspects of pet lemur ownership could change with human population and tourism (Table 1).

**Table 1:** Hypotheses on how the first and second aspects of pet lemur ownership could change with human population and tourism.

Number	Hypothesis	Rationale
1a	Pet lemur ownership is a national phenomenon and rates of ownership do not differ across the country	Pet lemurs appear to be owned by households across the country [5]. Because reasons for ownership differ [9] and because many pet lemurs are not kept in areas visible to the general public (e.g. not kept for tourists or others to see [17]), we hypothesize that rates of ownership do not broadly differ by province across the country, even if there are areas that might seem like ‘hotspots’ simply because lemurs in those areas are highly visible.
1b	Proportion of people who report owning lemurs increases as town population increases	Rates of pet lemur ownership have been hypothesized to increase as town population increases [5].
1c	Proportion of people who report owning lemurs does not increase with tourism	Because pet lemurs are owned for a multitude of reasons [9], we hypothesize that rates ownership do not increase with rates of tourism, even as tourism-related income is a driver to pet lemur ownership. Instead, we hypothesize that the visibility of pet lemurs, not the proportion of lemur owners in a community, is what changes as tourism rates increase (hypothesis 2c).
2a	Proportion of people who report seeing pet lemurs differs across the country	There is some evidence that there are ‘hotspots’ for areas where many people report seeing pet lemurs in Madagascar [13], even as it remains unclear whether this increase in pet lemur sightings is because of increased visibility of pets or because of increase numbers of lemurs kept as pets.
2b	The proportion of people who report seeing pet lemurs does not change with town population	Town population is not linked to the proportion of people in Madagascar who report seeing lemurs as pets [5]. In other words, people in small towns are just as likely to have seen a lemur as a pet as people in large towns.
2c	The proportion of people who report seeing pet lemurs increases as tourism increases	There are ‘hotspots’ where pet lemurs appear to be more visible [13], but these areas do not appear to be linked to a town’s human population [5]. Lemurs kept in visible locations where they might easily be seen are often those that are serving the tourism industry [9].

Understanding how the rates pet lemur ownership and openness with which people keep these animals overlap, is important for understanding how to combat illegal pet lemur ownership in Madagascar. This is because areas that have high rates of hidden pet lemur ownership may require different outreach and enforcement mechanisms than areas with more visible ownership. In addition, areas with a large number of pet lemurs, may not be towns with a high rate of lemur ownership or a highly visible pet lemur problem. We base our analysis on a national survey effort in 11 of Madagascar’s 22 administrative regions (in all of the country’s six former provinces, a previously used sub-national administrative boundary). Worldwide, there have been only a few nationwide surveys to examine the incidence of wild animal pet ownership (e.g. in Costa Rica [18]) and, to our knowledge, none have focused exclusively on primates in their endemic countries (though there have been several, substantial surveys at the sub-national level in different parts of the world). National surveys can provide an important baseline for understanding trends in illegal primate ownership, particularly for species that are already facing other threats, such as habitat degradation and hunting.

## Methods

### Ethical research considerations

International standards for research ethics were followed and research was approved by an ethics oversight committee (Institutional Review Board, Temple University in 2013; Institutional Review Board, University of Utah in 2016). Research followed all national and local laws pertaining to the survey of adults in Madagascar. Research was authorized by locally elected officials in every town and commune in which research took place. In 2013, as interviews were sometimes conducted along the perimeter of a national park, research was authorized by the Madagascar Ministry of Water and Forests and Madagascar National Parks. In 2016, this research required no government research permits.

### Legality of pet lemur ownership

It is illegal to keep lemurs in captivity in Madagascar without government-issued permits [19]. Under domestic law, the penalties for capturing, holding, buying, or selling a lemur include the confiscation of the lemur, a fine (10,000,000 Ariary to 50,000,000 Ariary), and/or imprisonment from 6 months to 2 years (Law N. 2005–018 dated 17^th^ October 2005, Republic of Madagascar). This domestic law aligns with international commitments. Madagascar is party to CITES (Convention on International Trade in Endangered Species of Wild Fauna and Flora [20]) and the CBD (Convention on Biological Diversity [21]) and has therefore agreed to regulate aspects of wild animal trade (and is encouraged to do so via national legislation and governance). There are very few individuals/entities (likely less than several dozen and certainly less than several hundred individuals/entities) that have the right to legally own a captive lemur in Madagascar by securing temporary permission (*Gardien séquestriel*) or longer-term permits (i.e. those granted to nature parks and zoos) from regional and national government bodies, respectively [19].

### Definition of pet lemurs

As per existing publications on this topic [5,9,13, 22], we define a pet lemur as being any lemur that is perceived to have a human owner (individual or business, regardless of the purpose of its captivity), has been removed from its natural habitat, or relies on humans for food. This can include lemurs that are habituated, restrained, and/or unrestrained. This does not include fully wild lemurs living on private lands or lemurs in zoos.

### Study area

**Fig 1:**
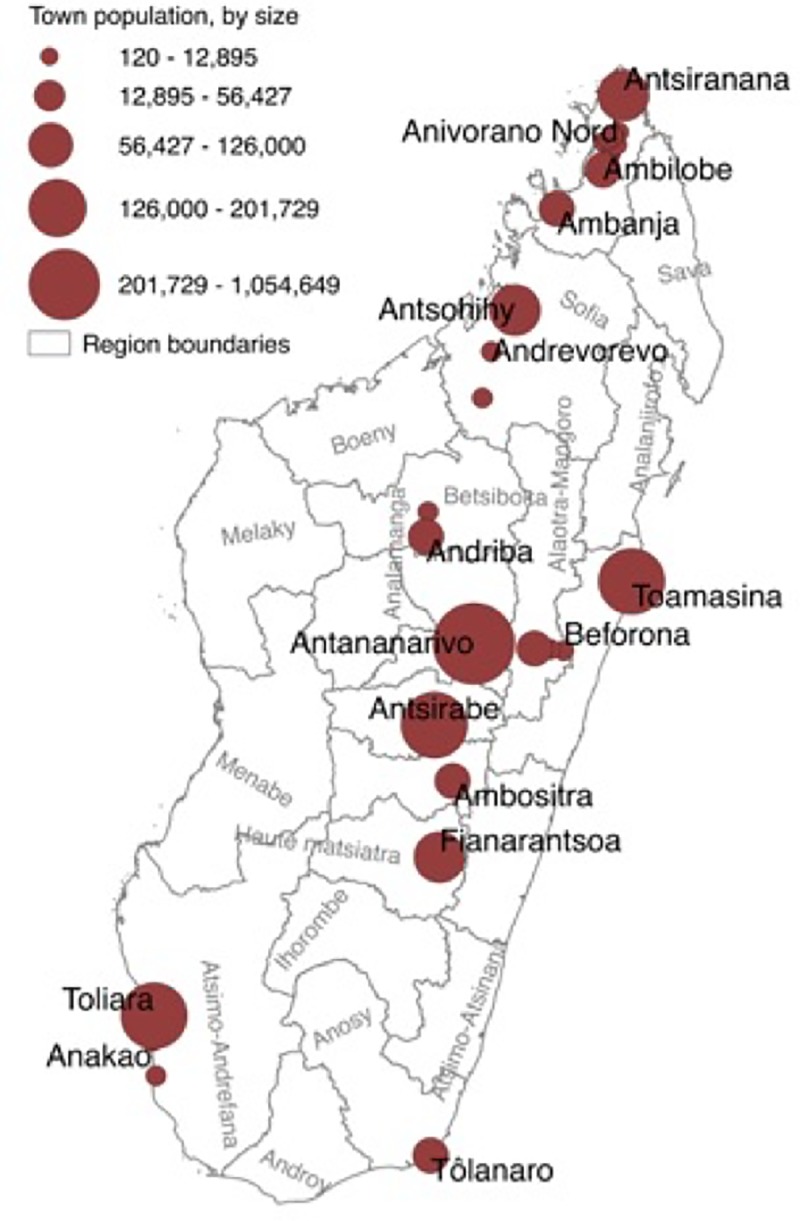
Map of towns included in our survey effort. Town names are indicated for towns in which there were 5,000 or more inhabitants. Circle size represents population size (circle size determined using Jenks natural breaks classification method). Regional boundaries are courtesy of the GADM dataset (http://gadm.org).

Data were collected from central, western, and northern Madagascar in June to August 2013 [4] and in central, southern, and eastern Madagascar in July to August 2016 [16] using semi-structured household interviews. In total, data were collected across 28 towns (Fig 1) via 1,709 household interviews (Table 2). These 28 towns included 9 towns with less than 5,000 inhabitants across in three administrative regions in the north, centre, and southern parts of the country. Based on Madagascar’s population of 24.9 million people [23] and mean household size (4.6 people per household averaging across rural and urban areas [24]), there are c. 5,413,043 households in the country. Our sample size (n = 1,709) is larger than the minimum number of household interviews (n = 1,067) required for our data to serve as an adequate sample in regards to the measure of national pet lemur ownership (*p* set to 50%; margin of error ± 3% of the estimate; 95% confidence level [25]).

**Table 2:** Towns included in this survey effort, as well as information regarding the characteristics of those towns.

Town	Province (based on prior government province boundaries)	Region	Town Pop	Number of people interviewed (one per household)	Percent (%) of households in the town interviewed	Number of photos posted to Flickr	Data source for pet lemur ownership/visibility data
Ambanja	Antsiranana	Diana	28,468	55	0.9	2,774	Reuter et al. 2016
Ambilobe	Antsiranana	Diana	56,427	99	0.8	1,308	Reuter et al. 2016
Ambondromifehy†	Antsiranana	Diana	5,000	30	2.6	3	Reuter et al. 2016
Ambositra	Fianarantsoa	Amoron'i Mania	31,818	99	1.4	3,287	This paper
Ampasinbengy†	Antsiranana	Diana	1,997	30	6.6	0	Reuter et al. 2016
Andranankoho†	Antsiranana	Diana	2,000	11	2.4	0	Reuter et al. 2016
Anakao*	Toliara	Atsimo-Andrefana	5,907 [26]	40	3.0	3,697	This paper
Andasibe	Toamasina	Alaotra-Mangoro	12,384	53	1.9	11,853	This paper
Andrevorevo^§^	Mahajanga	Sofia	7,512	40	2.3	2	Reuter et al. 2016
Andriba	Mahajanga	Betsiboka	32,000	74	1.0	20	Reuter et al. 2016
Aniverano Nord	Antsiranana	Diana	6,622	90	6.0	87	Reuter et al. 2016
Antsohihy	Mahajanga	Sofia	105,317	60	0.3	43	Reuter et al. 2016
Antananarivo	Antananarivo	Analamanga	1,054,649	253	0.1	28,330	Reuter et al. 2016 (n = 199); This paper (n = 54)
Antsiafabositra	Mahajanga	Betsiboka	8,328	69	3.7	0	Reuter et al. 2016
Antsirabe	Antananarivo	Vakinan-karatra	186,253	51	0.1	9,773	This paper
Antsiranana (Diego Suarez)	Antsiranana	Diana	87,569	180	0.9	15,784	Reuter et al. 2016
Beforona	Toamasina	Alaotra-Mangoro	12,895	54	1.8	83	This paper
Efoetsy**	Toliara	Atsimo-Andrefana	1,294 [27]	9	3.1	28	This paper
Fianarantsoa	Fianarantsoa	Haute Matsiatra	126,000	84	0.3	7,193	This paper
Tôlanaro (Fort Dauphin)	Toliara	Anosy	46,298	50	0.5	6,100	This paper
Lambondry†	Antsiranana	Diana	120	34	100.0	0	Reuter et al. 2016
Marotaolana†	Antsiranana	Diana	175	30	75.0	0	Reuter et al. 2016
Matzaborimanga†	Antsiranana	Diana	400	19	21.0	0	Reuter et al. 2016
Moramanga	Toamasina	Alaotra-Mangoro	40,050	60	0.7	1,392	This paper
Toamasina (Tamatave)	Toamasina	Atsinanana	201,729	50	0.1	6,396	This paper
Tsarakibany†	Antsiranana	Diana	250	30	52.8	0	Reuter et al. 2016
Toliara (Tulear)	Toliara	Atsimo-Andrefana	195,904	23	0.1	18,692	This paper
Tsararivotra^§^	Mahajanga	Sofia	2,232	32	6.3	0	Reuter et al. 2016
TOTAL			2,259,598	1709	0.3	116,845	

Where towns have been historically called by a different, French name, the name is provided in parentheses. The percentage of households in the town interviewed, is on the assumption that there are 4.4 people per household [24]. Data were collected in 2013 and 2016, with data collected in 2013 initially published in Reuter et al. 2016 and data collected in 2016 published in this paper for the first time.

* Town population estimate from a 2007 government census.

** Town population estimate based on 205 households or 1294 inhabitants.

† Population data provided to researchers in 2013 by town-level elected officials.

§ Estimated by researchers by approximating the number of buildings within town boundaries using satellite imagery (on google maps) and multiplying by five (assuming 5 individuals per household). We then rounded up by 25% to address the undercounting that occurred due to the low quality of the satellite imagery. We recognize that this may over- or under-estimate true population.

In 2013, urban and rural towns were surveyed at relatively regular intervals along a 1,092-km highway ‘transect’ along the RN4/RN6 roads linking Antananarivo (central Madagascar) to Antsiranana (northern Madagascar). Rural towns were also surveyed on the perimeter of the Ankarana National Park, including several towns with no road access. In 2016, urban towns were selected at regular intervals for surveying along a 747-km highway ‘transect’ beginning in Toamasina (eastern Madagascar) and going central/south via the RN2/RN7 roads down to the town of Fianarantsoa. In addition, in 2016, several urban and rural towns in the southern province of Toliara were sampled (including Tôlanaro, Toliara, and smaller towns in the vicinity of these urban centers); these were not sampled using our overland, ‘transect’ approach due to safety concerns regarding travel by car.

The towns selected in 2013 and 2016 for surveying included: 1) six of the seven largest towns in Madagascar (from largest to smallest: Antananarivo, Toamasina, Antsirabe, Fianarantsoa, Toliara, Antsiranana) as well as nine small towns (120 to 5,000 people) in three different regions of the country; 2) areas where it was suspected that pet lemur ownership would be popular (smaller, more rural towns in the Toliara province; larger, more urban towns such as Toamasina and Tôlanaro); and 3) areas where it was not suspected that pet lemur ownership would be popular (smaller, more rural areas around the Ankarana National Park; in the capital city of Antananarivo). Towns are located in 11 of Madagascar’s 22 administrative regions and all of Madagascar’s six former provinces (a sub-national administrative unit phased out in 2009), in rural and urban areas, adjacent and distant from protected areas, with at least nine different primary ethnicities and four secondary ethnicities found across the towns. The combined human population of the 28 towns surveyed was approximately 2.26 million people (out of 24.9 million people nationwide [23]) with approximately 510,484 households in the 19 towns where we undertook surveys in which the town population surpassed 5,000 people.

Efforts were made to ensure representation of a wide range of urban and rural areas as well as to ensure that towns surveyed represented of a diversity of attributes that could affect rates of pet lemur ownership (e.g. road access, proximity to protected areas, respondent ethnicity). As with any extrapolation effort, however, biases are likely. For example, additional surveys in rural areas or different administrative regions may have yielded different pet lemur ownership rates and affected our estimates on national rates of pet lemur ownership. The authors acknowledge that the survey effort could have excluded areas in Madagascar that were hotspots for pet lemur ownership but note that surveys were done in the three administrative regions (out of 22) where pet lemur ownership has been hypothesized to be at its highest [13].

### Household surveys

The same data collection protocols were used in 2013 and 2016, except where noted, below. In both years, verbal informed consent was received and interviews were conducted by a two-person team comprised of one international project leader and one trained Malagasy translator. The decision to undertake interviews using a two-person team with one non-Malagasy person present, followed a number of pilot interviews in 2013 where it was found that interviewee trust in the survey process as well as rates of reported sightings of illegal pet lemurs tended to increase when a non-Malagasy interviewer was present. This appeared to be for numerous reasons, the most important being reassurance that information provided during the survey would not be reported back to any Malagasy authorities.

We used random sampling stratified by administrative unit with 5–10 interviews conducted across as many different communes/quarters within each town as time would allow. In smaller towns, we sampled every fifth household; in some cases we sampled virtually all households in the town (Table 1). The percent of household sampled within a town ranged from an estimated 0.1% to 100% (11.5% ± 27.9%, towns as replicates, mean ± 95% CI). However, the variability in the proportion of households interviewed in towns with human populations of 5,000 or more, was lower at 1.4% ± 1.5% of households sampled (range: 0.05–6.0%, towns as replicates). To ensure independent sampling, only one adult was interviewed per household. If an eligible individual refused to participate or if nobody was present, sampling continued at the next household. Interviews were anonymous and no identifying information was collected. We did not collect socioeconomic information for respondents (e.g. religion, ethnicity, education level). Interviewees were not compensated for their participation in the survey.

In both years, interviewees were asked whether they had ever seen a captive lemur, using locally appropriate words for ‘pet’ and if so, we asked follow-up questions. Interviews were always conducted in the local dialect and/or the interviewee’s preferred dialect. In both years, we asked these follow-up questions: Where, when and how many did you see? In addition, in 2016, we also asked a series questions regarding the motivations and captive conditions of ownership (the results of which are not covered in this paper). In 2016, but not in 2013, we asked individuals whether they were the owner of the lemur that they were reporting to us. In 2013, some interviewees self-reported owning lemurs even though we did not prompt them to provide us with this information. Despite the difference in collecting information about whether or not the respondent was a current/former owner of a pet lemur, there was no difference in the proportion of respondents who self-reported being current or former owners of pet lemurs in 2013 and 2016 (Kruskal Wallis Rank Sums Test, Chi-Square = 2.4229, DF = 1, P = 0.1196).

We did not provide interviewees with a definition of a ‘pet lemur’ [5] though the researchers’ definition of what constitutes a pet lemur can be found, above. We excluded lemurs seen in zoos or reserves.

### National analysis of pet lemur ownership in Madagascar

In this study, we aimed to better estimate the number of lemurs kept as illegal household pets in the recent past at a national level. In addition, we were interested in examining three different aspects of pet lemur ownership: rates of ownership, the visibility of pet lemur ownership, and the absolute number of lemur owners in an area.

In order to estimate the total number of pet lemurs in Madagascar in the recent past, we extrapolated the frequency of lemur ownership (following [4]). We conservatively assumed that only one lemur was owned per individual, who represented one household. We did not exclude pet lemur owners who had owned lemurs in a town that was different from the one in which they were interviewed (i.e. in the town that they resided in at the time of the interview). We present the estimated number of lemurs owned by households in Madagascar at the national scale in the recent past (3 years, 1.5 years, and 0.5 years prior to the survey). Based on Madagascar’s population of 24.9 million people [21], and mean household size (4.6 people per household averaging across rural and urban areas [22]), there are c. 5,413,043 households in the country. We also present the estimated number of lemurs owned by urban households in Madagascar at the national scale in the recent past (3 years, 1.5 years, and 0.5 years prior to the survey); in this case, we extrapolated from the frequency of lemur ownership from our survey towns that had human populations of greater than 5,000 people. Based on Madagascar’s urban population (8,926,000 people in 2016 [28]) and mean urban household size (4.4 people per household [24]), there are c. 2,028,636 urban households in the country. Finally, we present the estimated number of lemurs kept as pets in the large towns (human population of more than 5,000 people, following [5]) where we conducted interviews (3 years, 1.5 years, and 0.5 years prior to the survey).

For the first aspect of ownership that was studied (rates of pet lemur ownership), the proportion of respondents that had owned a lemur (in their lifetimes and within the 1.5 years prior to survey administration) was a proxy for identifying areas with high rates of lemur ownership. Here, we excluded people who had owned a lemur in a town that was different from the one in which they were interviewed in (i.e. a town in which they did not reside). This was done because numerous respondents told us information about lemurs that they had owned in areas of Madagascar where we had not conducted surveys and because of how the data were collected, we could only systematically assess rates of ownership within towns where we conducted interviews. Most owners (69 ± 13%, n = 18 towns) who we spoke to, had owned their lemurs in the same towns where they were interviewed.

For the second aspect of ownership studied (visibility of pet lemur ownership), the proportion of respondents that had seen a pet lemur (in their lifetimes and within the 1.5 years prior to survey administration) in each town was a proxy for identifying areas where pet lemur ownership is more visible or less concealed. We excluded people who had seen a pet lemur in a town that was different from the one in which they were interviewed in (i.e. a town in which they did not reside). This was done because numerous respondents told us information about lemurs that they had seen in areas of Madagascar where we had not conducted surveys (and we could not accurately calculate the frequency of respondents seeing pet lemurs in towns where we did not conduct our survey efforts). About half of the lemurs that people had seen (49 ± 11%, n = 25 towns), were seen in the same towns where they were interviewed.

For the third aspect of ownership studied, we multiplied the estimated number of households per town by the percent of people who had owned captive lemurs in those towns; this provided us with an estimate of the absolute number of people within a town that were current of former pet lemur owners and provides a different perspective on pet lemur ownership than the first aspect we examined. For example, it is important to know not only which town has a high proportion of pet lemur ownership but also which town, because of its human population, would have the highest absolute number of owners. It is conceivable that a town with very low rates of pet lemur ownership could actually have a high number of pet lemur owners, simply because it has a high human population.

For the first two aspects of ownership that were studied, we examined several hypotheses (Table 1), including: 1) whether there was regional variation across the country (using towns as replicates within Madagascar’s six provinces); 2) whether there is support for prior conclusions [5] regarding a link between an increase in ownership with the population of a town; and 3) whether there was a link with the popularity of the town with tourists, using the number of photos geo-tagged to the town on Flickr (a popular photo-sharing website, www.flickr.com). We did not analyze the link between pet lemur ownership and suspected drivers of ownership such as fear of enforcement and social status, due to a lack of data collected on these topics during our surveys (and a lack of town-level proxy data available). For the third aspect of pet lemur ownership, the town-level metrics are simply a multiplication of the rate of ownership (presented as part of the first aspect) by the number of households in a town.

It should be noted that the Government of Madagascar ceased using ‘provinces’ as a sub-national administrative unit in 2009 (the highest sub-national administrative unit now being the 22 administrative regions in the country). However, we sometimes analyze our data using towns as replicates within the (former) province boundaries because: 1) the number of towns sampled would be too low to adequately represent each of the 22 administrative regions (or even each of the 11 administrative regions in which the towns are found); 2) the six (former) provinces provide a sub-national unit that is sufficiently large for our town-level data to be considered reasonably representative for each province.

### Study species

In both 2013 and 2016, our questions solicited responses regarding all lemur species. In 2013, we did not collect genus- or species-level data in a robust manner (see [5]); it quickly became clear during data collection that common names were often being used to describe multiple species and that most interviewees were not able to provide any robust information related to species descriptions at all.

In 2016, when collecting data in central and southern Madagascar, we used approximately 12 different photos of lemurs to ensure they reported pet ownership of a range of lemur species (e.g. including *Microcebus* spp. which we suspected were being underreported because they do not look like a ‘typical’ lemur) and to solicit some genus-level and species-level data. Given that there are over 100 lemur species, we chose not ask respondents to identify pet lemurs to the species, but used the photos to help prompt their memory while providing us with some genus-level information. It is important to mention that to the untrained eye, many lemur species look phenotypically similar. Most people in the Malagasy general public, particularly those in urban areas who have only seen someone else’s pet lemur in passing, would not be able to distinguish–for example–two *Eulemur* spp. from each other in photographs. With these caveats noted, we present the genus and species-level data here. For context, 30 species of lemur have been recorded in captivity in Madagascar [13].

Due to sample size issues, we only present estimated rates and magnitude of ownership for *Lemur catta*. Magnitude of ownership is calculated simply by multiplying the percent of household ownership in each town by the number of households in that town (assuming one household owned only one lemur). For *Lemur catta*, we do not extrapolate to other towns not surveyed, given the regional nature of these data.

### Analysis

As there may be greater variation between than within study sites, towns are used as replicates for most analyses. Results are presented as mean values with 95% confidence intervals.

For analyses aiming to examine the link between aspects one and two and tourism, we used the number of photos geo-tagged to towns on Flickr as a proxy for the popularity of a town in regard to tourism. Flickr is a popular photo-sharing website for travel photos (www.flickr.com). Flickr has been used in numerous studies, including as a proxy to quantify nature-based tourism and recreation [29] and the contribution of IUCN Red List species to nature-based tourism in Africa [30]. Flickr is used as proxy for tourism as it includes photos posted by both overnight and day-trip tourists and allows for comparable statistics among towns that are otherwise vastly different (e.g. small towns around protected areas that have few hotel rooms but a large number of day-trip visitors can be compared against large towns, like Antananarivo, that have a large number of overnight visitors but not usually for wildlife-related tourism). It was not possible to use other types of tourism-related information at the town-level (e.g. such as the number of hotels within a town) as this information was not collected in our 2013 survey efforts; publicly available reports on Madagascar’s tourism facilities are relatively outdated and/or do not present information at the town-level (e.g. [31]). As would be expected given that we surveyed in areas that were both popular and not popular with domestic/international tourists, the number of photos posted to Flickr differed widely by town (non-normal distribution, Table 2); therefore, the number of photos were natural log-transformed prior to analysis. The number of photos posted to Flickr for a town increased with the population of the town (Regression, F-Ratio = 50.7026, R^2^ = 0.648, N = 28, P < 0.0001).

For regression analyses including town populations as the dependent variable, town population was natural log-transformed prior to analysis due to non-normal distributions (following [5]).

## Results

### Data parameters

One-third (36% ± 10%, towns as replicates) of respondents had seen a captive lemur in their lifetimes, and 4% ± 2% had personally owned one (currently or at any time in the past). The percentage of people in a town who had seen a pet lemur did not correlate with the percentage of people in a town who had owned a lemur (Regression, F-Ratio = 0.5343, R square = 0.020, N = 28, P = 0.4713). In other words, the towns in which a higher proportion of people had owned a lemur were not the same towns in which a higher proportion of interview respondents had seen pet lemurs. In 2016, 59% of 582 respondents interviewed were women and 41% were men. All respondents were legal adults over the age of 18.

### Magnitude of lemur ownership

We estimate that 33,428 ± 24,846 (mean ± 95% CI) lemurs were kept across households in Madagascar in the six months prior to our survey efforts (Table 3). At a sub-national level, we estimate that in the six months prior to our survey efforts, there were 18,462 ± 12,963 pet lemurs in urban households across the country (or 43,208 ± 18,704 in urban households in the 3.5 years prior to survey efforts) of which 4,646 ± 3,262 pet lemurs were found in the large towns (town population of at least 5,000 people) where we conducted our surveys.

**Table 3:** Estimated magnitude of pet lemur ownership in Madagascar.

	Number of Households	Percent of households owned lemurs in 3.5 years prior	Number of households owned lemurs (3.5 years prior)	Percent of households owned lemurs in 1.5 years prior	Number of households owned lemurs (1.5 years prior)	Percent of households owned lemurs in 0.5 years prior	Number of households owned lemurs (0.5 years prior)
Number of lemurs owned by households nationally	5,413,043 (number of households in Madagascar)	1.5% ± 0.7% (n = 28 towns)	78,234 ± 39,190	1.0% ± 0.5% (n = 28 towns)	55,909 ± 27,552	0.6% ± 0.5% (n = 28 towns)	33,428 ± 24,846
Number of lemurs owned by households in urban towns (over 5,000 people) nationally	2,028,636 (number of urban households in Madagascar)	2.1% ± 0.9% (n = 19 towns)	43,208 ± 18,704	1.5% ± 0.6% (n = 19 towns)	30,878 ± 12,963	0.9% ± 0.6% (n = 19 towns)	18,462 ± 12,963
Number of lemurs owned by households in large towns (over 5,000 people) surveyed in this study	510,484 (number of households in towns surveyed over 5,000 people)	2.1% ± 0.9% (n = 19 towns)	10,873 ± 4,707	1.5% ± 0.6% (n = 19 towns)	7,770 ± 3,288	0.9% ± 0.6% (n = 19 towns)	4,646 ± 3,262

### Rates of lemur ownership (aspect one of lemur ownership)

The proportion of respondents that self-reported having ever owned a lemur in the same town where they were surveyed ranged from 0% to 10% of respondents per town (Table 4). The proportion of respondents that reported having owned a lemur in the same town in which they were surveyed, in the 1.5 years prior to the survey, ranged from 0% to 4.4% (Table 4). Additional data on the rates of ownership can be found in the supplementary materials (S1 Table).

**Table 4:** Rates of pet lemur ownership and visibility of pet lemur ownership.

Town	Province	Percentage of respondents who had owned a pet lemur*		Percentage of respondents who had seen a pet lemur*	
		Anytime in the past	In the 1.5 years prior to being surveyed	Anytime in the past	In the 1.5 years prior to being surveyed
Ambanja	Antsiranana	5.5%	0%	38%	16%
Ambilobe	Antsiranana	3.0%	0%	16%	8%
Ambondromifehy	Antsiranana	0%	0%	0%	0%
Ambositra	Fianarantsoa	5.1%	2.0%	25%	17%
Ampasinbengy	Antsiranana	0%	0%	17%	7%
Andranankoho	Antsiranana	0%	0%	0%	0%
Anakao	Toliara	0%	0%	33%	3%
Andasibe	Toamasina	3.8%	1.9%	6%	0%
Andrevorevo	Mahajanga	2.5%	2.5%	23%	13%
Andriba	Mahajanga	4.1%	0%	3%	1%
Aniverano Nord	Antsiranana	0%	0%	27%	19%
Antsohihy	Mahajanga	5.0%	1.7%	45%	0%
Antananarivo	Antananarivo	0.4%	0%	10%	4%
Antsiafabositra	Mahajanga	4.4%	0%	0%	0%
Antsirabe	Antananarivo	3.9%	2.0%	22%	4%
Antsiranana (Diego Suarez)	Antsiranana	2.8%	1.1%	14%	6%
Beforona	Toamasina	1.9%	0%	2%	0%
Efoetsy	Toliara	0%	0%	11%	0%
Fianarantsoa	Fianarantsoa	3.6%	0%	27%	11%
Tôlanaro (Fort Dauphin)	Toliara	10.00%	4.0%	50%	36%
Lambondry	Antsiranana	0%	0%	0%	0%
Marotaolana	Antsiranana	0%	0%	0%	0%
Matzaborimanga	Antsiranana	0%	0%	0%	0%
Moramanga	Toamasina	3.3%	0%	0%	0%
Toamasina (Tamatave)	Toamasina	0%	0%	26%	8%
Tsarakibany	Antsiranana	0%	0%	7%	3%
Toliara (Tulear)	Toliara	4.4%	4.4%	61%	43%
Tsararivotra	Mahajanga	0%	0%	19%	3%
TOTAL		1.1 ± 0.7%	0.7 ± 0.5%	18 ± 6%	8 ± 4%

Pet lemur ownership rates shown with one significant digit due to the low percentage rates. Data for towns with a population of less than 5,000 people are omitted (marked with ‘OM’) to protect respondent anonymity. Additional data on rates of pet lemur ownership can be found in the supplementary materials (S1 Table).

*Only considering respondents who had owned or seen a lemur in the same town in which they were interviewed.

In accordance with hypothesis 1a, it appears that ownership is rare but nevertheless found to occur across Madagascar. The top five towns with the highest rates of pet lemur ownership (i.e. towns with the highest percentage of respondents reported having owned a pet lemur) came from four different provinces (Table 4). The proportion of respondents who had ever owned a captive lemur did not differ by province (Kruskal-Wallis Rank Sums Test, Chi-square = 6.9331, DF = 5, P = 0.2257, towns as replicates). Likewise, the proportion of respondents who had owned a lemur in the same town in which they were surveyed, in the 1.5 years prior to our survey, did not differ by province (Kruskal-Wallis Rank Sums Test, Chi-square = 5.3938, DF = 5, P = 0.3697, towns as replicates).

There was mixed evidence for hypothesis 1b. There was an increase in the proportion of respondents who had ever owned a captive lemur as the population of a town increased (considering only respondents who owned a pet lemur in the same town in which they were interviewed; Regression, F-Ratio = 9.8438, R^2^ = 0.275, N = 28, P = 0.0042, population log-transformed). However, there was no increase in the proportion of respondents who had owned a pet lemur in the last 1.5 years with the population of a town (considering only respondents who owned a pet lemur in the same town in which they were interviewed; Regression, F-Ratio = 4.2096, R^2^ = 0.139, N = 28, P = 0.0504, population log-transformed). The top five towns with the highest rates of pet lemur ownership ranged widely in size both when considering lifetime ownership (range: 8,328–105,317 people; Tables 2 and 5) and when considering rates of pet lemur ownership in the 1.5-year time period prior to surveys (range: 7,512–195,904 people; Tables 2 and 5).

**Table 5:** The top 5 towns (‘hotspots’) as categorized under the three different components discussed in this paper.

Town	Province	Percentage of respondents who had owned a pet lemur*		Percentage of respondents who had seen a pet lemur*		Estimated number of households owning a pet lemur*	
		Anytime in the past	In the 1.5 years prior to being surveyed	Anytime in the past	In the 1.5 years prior to being surveyed	Anytime in the past	In the 1.5 years prior to being surveyed
Anakoa	Toliara			5			
Andrevorevo	Mahajanga		3				
Anivorano Nord	Antsiranana				4		
Antsirabe	Antananarivo		5			2	2
Antsiranana (Diego Suarez)	Antsiranana						5
Antsohihy	Mahajanga	4		3	3	3	4
Ambanja	Antsiranana	2		4			
Ambositra	Fianarantsoa	3	4		5		
Fianarantsoa	Fianarantsoa					5	
Toliara (Tulear)	Toliara	5	1	1	1	1	1
Taolagnaro (Fort Dauphin)	Toliara	1	2	2	2	4	3

These hotspots exclude towns that we surveyed in which there were less than 5,000 inhabitants to protect anonymity. Towns are also not listed if they did not rank as one of the top 5 towns for any of the categories. Towns are ranked from 1 to 5, with lower numbers indicating a higher-ranking relative to other towns.

*Only considering respondents who had owned or seen a lemur in the same town in which they were interviewed.

In contrast to hypothesis 1c, there was an increase in the proportion of respondents who had owned a captive lemur with the popularity of a town on Flickr (considering only respondents who owned a pet lemur in the same town in which they were interviewed; Regression, F-Ratio = 7.8797, R^2^ = 0.233, N = 28, P = 0.0094, number of photos geographically linked to the town log-transformed, population of town log-transformed). Likewise, there was an increase in the proportion of respondents who had owned a captive lemur with the popularity of a town on Flickr in the 1.5 years prior to the survey (considering only respondents who owned a pet lemur in the same town in which they were interviewed; Regression, F-Ratio = 5.0608, R^2^ = 0.163, N = 28, P = 0.0332, number of photos geographically linked to the town log-transformed, population of town log-transformed).

### Proportion of people who had seen pet lemurs (aspect two of lemur ownership)

The proportion of respondents that reported having seen a pet lemur in the towns where they were interviewed, ranged from 0% to 61% of respondents per town (Table 4). The proportion of respondents that saw a pet lemur in the same town in which they were surveyed, in the 1.5 years prior to the survey, ranged from 0% to 43% (Table 4).

In contrast with hypothesis 2a, the proportion of people who had seen a pet lemur did not differ across the country, though three of the five top towns where respondents had ever seen lemurs (i.e. the highest percentage of respondents had seen a pet lemur), were in the Toliara Province (Table 5). However, in the 1.5 years prior to the survey, four provinces were represented by the top five towns where respondents had seen pet lemurs (Table 5). The proportion of respondents who had ever seen a captive lemur in their towns of residence did not differ by province (considering only respondents who saw a pet lemur in the same town in which they were interviewed; Kruskal-Wallis Rank Sums Test, Chi-square = 7.9367, DF = 5, P = 0.1598, towns as replicates). Likewise, the proportion of respondents who had seen a pet lemur in the 1.5 years prior to the survey, did not differ by province (considering only respondents who saw a pet lemur in the same town in which they were interviewed; Kruskal-Wallis Rank Sums Test, Chi-square = 3.7415, DF = 5, P = 0.5872, towns as replicates).

In contrast with hypothesis 2b, there was an increase in the proportion of respondents who had ever seen a captive lemur in their town of residence as the population of a town increased (Regression, F-Ratio = 9.7567, R^2^ = 0.2729, N = 28, P = 0.0044, population of towns log-transformed). Likewise, there was an increase in the proportion of respondents who had ever seen a pet lemur in their town of residence in the 1.5 years prior to the survey, as the population of a town increased (Regression, F-Ratio = 6.5431, R^2^ = 0.2011, N = 28, P = 0.0167, population of towns log-transformed).

In accordance with hypothesis 2c, there was an increase in the proportion of respondents who had ever seen a captive lemur in their town, with the popularity of a town on Flickr (Regression, F-Ratio = 10.9072, R^2^ = 0.2955, N = 28, P = 0.0028, number of photos geographically linked to the town log-transformed, population of towns log-transformed). Likewise, there was an increase in the proportion of respondents who had seen a captive lemur in their town, in the 1.5 years prior to the survey, with the popularity of a town on Flickr (Regression, F-Ratio = 5.5123, R^2^ = 0.1749, N = 28, P = 0.0268, number of photos geographically linked to the town log-transformed, population of towns log-transformed).

### Areas where high numbers of pet lemurs could be kept as pets (aspect three of lemur ownership)

Four of the five highest-ranking towns (i.e. compared to all of the towns surveyed in this paper) identified here, had already been identified as important towns under the prior two components (Table 5). However, the ranking of the towns differed across the three different areas of our analysis, highlighting the importance of weighing distinct criteria when identifying areas as ‘hotspots’ for ownership.

### Species of pet lemur owned

Of the 627 households interviewed in 2016 in central and southern Madagascar, 271 reported having seen a pet lemur (Table 6). The most commonly reported species seen as a pet were *L*. *catta* (49.4% of respondents) with *Eulemur* spp. being the only other group of lemurs reported by a substantial proportion of respondents.

**Table 6:** Genus and species (when known) of lemurs seen as pets by 271 households interviewed in 2016 in central and southern Madagascar.

Genus and/or species	Number of households (% of households)
*Allocebus* spp.	1 (< 1%)
*Avahi* spp.	2 (< 1%)
*Cheirogaleus* spp.	9 (3.3%)
*Eulemur* spp.	56 (20.7%)
*Hapalemur* spp.	17 (6.3%)
*Indri indri*	2 (< 1%)
*Lemur catta*	134 (49.4%)
*Lepilemur* spp.	3 (1.1%)
*Microcebus* spp.	6 (2.2%)
*Mirza* spp.	4 (1.5%)
*Prolemur simus*	9 (3.3%)
*Propithecus* spp.	8 (3.0%)
*Varecia* spp.	9 (3.3%)
Unable to identify from photographs	14 (5.2%)
Total	271

Some households reported seeing more than one type of genus/species.

Given the uncertainty with respondent recall, the genus and species identifications should be treated with caution. However, it does appear some were correctly identified. For example, *Hapalemur* spp. were reported by respondents only in towns near/within the species’ ranges (S2 Table). *L*. *catta–*a visually distinctive species due to its characteristically ringed tail–were seen as pets by households in every town where interviews were conducted in 2016, though they were reported less by households outside the species’ range (S2 Table).

*L*. *catta* was the only genus/species with a sample size large enough to estimate rates of ownership (n = 8 households reporting having owned one in 3.5 years prior to interview, across 7 towns where interviews took place in 2016). In the 12 towns surveyed, 1.5% ± 1.4% (mean ± 95% C.I, towns as replicates) of households had owned a *L*. *catta* in the 3.5 years prior to interview. This decreased to 1.2% ± 0.9% of households surveyed in central and southern Madagascar in the 1.5 years and 0.5 years prior to interview.

We estimate that in the towns where we conducted interviews in 2016, the magnitude of *L*. *catta* ownership was as follows: 1) 3,790 individuals kept as pets by households in the 12 towns we surveyed in the 3.5 years prior to interview; or 2) c. 3,369 individuals kept as pets in the 12 towns surveyed in the 1.5 years and 0.5 years prior to interview.

## Discussion

To our knowledge, this study is the first nationwide survey which focuses solely on primate pet ownership prevalence in a country where the primates are endemic. In addition, this study is novel in its approach to try and disentangle whether areas with high rates of pet primate ownership are also the areas where pet primates are highly visible and where the highest absolute number of pet primate owners live.

### Magnitude of lemur ownership

It was previously estimated that 28,253 lemurs had been kept as pets in urban areas during a 3.5-year period from 2010 to mid-2013 [5]. In this study, which includes these prior data [5] but expands the dataset to a more nationally-representative sample, we estimate that 43,208 ± 18,704 (mean ± 95% CI) lemurs were kept as pets in urban households in the 3.5 years prior to survey efforts. This estimate is not significantly different than the prior estimates [5]. Perhaps more relevant for conservation efforts, however, this study was able to estimate the number of lemurs kept, nationally, as pets over a six-month pre-survey period (33,428 ± 24,846 lemurs), thanks to the larger sample size that a national survey afforded the researchers.

The rate of pet lemur ownership did not differ by province and is low; only 4% ± 2% of households in Madagascar are estimated to have ever owned a lemur. Even still, and as evidenced by the estimates presented in this paper, the prevalence of pet lemur ownership should be considered an important tertiary threat to some lemur species, in the context of ongoing habitat degradation and hunting [10]. Many lemurs are not successfully kept as pets for a more than a few months [5], either because the species simply cannot survive on a captive or translocated diet (e.g. many Indriidae or Lepilemuridae, [15,16]), poor captive conditions [20], or because they are wild captured well before they are weaned from their mothers [12]. One-third of attempts at owning pet lemurs ends with the death of the lemur [32]. Once lemurs are kept as pets, it is virtually impossible to return them to the wild [19]. There are instances when pet lemurs are ‘freed’ by owners back into the wild [32] but we have no data on the survival rates of these informal reintroduction attempts. As such, most of the 33,428 ± 24,846 lemurs that we estimated were kept as pets across Madagascar in the six months prior to our survey, probably died within a few months of being extracted from the wild and–even when they survived–will not be returned successfully to the wild.

### Rates of lemur ownership

It is increasingly clear that a small but consistent minority of individuals in Madagascar are keeping lemurs as pets. Ownership rates do not appear to differ geographically (aspect one). Though it was previously hypothesized that rates of ownership increased with human population [5], this study evidences that this may not be the case. Interestingly, a novel finding of this paper is that the rates of pet lemur ownership do not correlate with the proportion of people who have seen pet lemurs in the same town. In addition, the rates of pet lemur ownership increased with the town’s popularity on Flickr, providing more evidence of the link between tourism and pet lemur ownership. However, as the explanatory power of the relationship is low (R^2^ = 0.233 and R^2^ = 0.163, respectively, for ownership rates at any point before survey and in the 1.5 years prior to the survey) and there are clearly other factors that are important in predicting rates of pet lemur ownership that remain to be examined (including various socioeconomic variables not collected by the authors of this paper; distance to protected areas, etc.). Certainly, it would be helpful in future studies to be able to fully consider species or species characteristics, though our paper provides the first estimates for sub-national rates of illegal pet lemur ownership for any lemur species (our estimates for *L*. *catta*).

### Visibility of pet lemur ownership

We did not find that the proportion of people seeing pet lemurs differed across the country, nor was it correlated with the rate of pet lemur ownership in a town. In other words, the towns where the highest proportion of people had seen lemurs were not the towns in which the highest proportion of people reported being owners of lemurs. Furthermore, we found that the residents of towns that were more popular with tourists (at least as measured using our proxy for tourism), regardless of whether the town had a small or large human population, were more likely to have seen a lemur as a pet.

The low visibility of pet lemur ownership in some areas of the country (Table 4) could explain why–despite thousands of lemurs being kept in illegal captivity across the country each year–some groups of people may never come across a pet lemur when living in, or visiting, Madagascar. For example, in towns that were not popular with tourists, it was not uncommon for respondents to be unaware of lemurs kept as personal pets in adjacent buildings (KER, pers. obs.), and this was usually the case when the lemurs were being kept in cages or in housing compounds with high walls (though in some cases, it was clear that respondents were withholding information from researchers, probably because they knew it was illegal to keep lemurs as pets). The challenges in collecting data about illicit, illegal, and informal trades are well known (reviewed by [7]). As another example, prior to 2015, there was low awareness among the scientific community that the ownership of pet lemurs was indeed happening at such a large scale in Madagascar (see [8,13]). Similar to other primate trades (e.g. [33]), scientists and researchers working in Madagascar did not generally report anecdotal observations of captive lemurs in their publications [13] nor was it widely recognized as a threat facing some lemur species (e.g., [10]). In addition, individuals who responded to the Pet Lemur Survey effort (a web-based survey of the public, but targeting the scientific community, about their interactions with pet lemurs in Madagascar [13]) had often only seen one (or a few) lemurs as pets, with some respondents commenting that they had never seen one despite years of working in Madagascar. This could be because scientists tend to not spend a lot of time in private households in large urban areas, where many pet lemur owners reside. The low visibility of pet lemurs in some areas is likely also due to the fact that the trade of live lemurs in Madagascar is extremely informal and not conducted in highly visible areas [5]. Captive lemurs are not sold through markets or established middlemen (or if they are, these entities are very well hidden and have not been discovered by the researchers despite a multi-year research effort). Even legal captive facilities in the country share very little information publicly about their captive lemur populations [19].

### Conservation implications

The outputs of our study highlight the need for an increased focus on the ownership of illegal pet lemurs. The town of Toliara was the only place where 10% of individuals surveyed had owned a lemur in Toliara any time in the past (though more than 10% of residents surveyed in the towns of Ambanja, Antsiafabositra, and Taolagnaro had owned lemurs in past elsewhere in the country). We therefore suggest the town of Toliara (and surrounding tourist sites) as a prudent target for conservation programming efforts. However, because conservation resources are limited, it is important to look at the different aspects of pet ownership that we examined, to ensure that towns are targeted not just because of the proportion of inhabitants that own pet lemurs but also the total number of lemurs that are potentially being kept as pets in those towns. This difference in targeted programming is highlighted in Table 5, where the towns with the highest proportion of lemur owners do not always overlap with the towns that would be expected to have the highest absolute number of individual pet lemurs. As a hypothetical example, a town with 500 houses in which 10% of houses owned a lemur would have just 50 lemurs in captivity while a town with 50,000 houses in which 3% of houses owned a lemur would have 1,500 lemurs in captivity. As such, and given the difficulty in individually targeting pet lemur owners who are keeping their lemur ownership hidden, conservation programming might consider large-scale outreach to the public on this issue.

Addressing areas where many people have seen illegal pet lemurs (examined under aspect two) will require a different approach used to address high rates of ownership (examined under aspect one). There are negative conservation consequences to highly visible illegal pet lemur ownership, which have been well-documented in the literature. For example, it is known that when Endangered animals–including *L*. *catta*–are seen in anthropogenic settings (such as in photographs), it can increase the incorrect perception that those animals are not Endangered [34]. As such, it is possible that the general public in areas where there are highly visible pet lemurs may be under the incorrect assumption that these animals have healthy populations in the wild.

Although there is room for more robust analyses examining the link between tourism and pet lemur ownership, we found that towns with increased tourism (using Flickr as a proxy) were more likely to have visible pet lemurs, and that the towns with the most visible pet lemurs were often located in the Toliara province. In these areas, there are typically a few, well-known entities (e.g., hotels, eco-lodges; not named here because of the illegal nature of ownership) that keep pet lemurs in such a way that is visible to the public (particularly tourists who view the lemurs as photo props or as an ‘added value’ attraction). Therefore, addressing this matter requires dealing with known lemur owners that are engaging in public, illegal behavior (and often being rewarded for that illegal behavior by receiving money or resources from tourists) and thereby being seen by the Malagasy general public as having higher social status or wealth [16]. We have anecdotally seen this to result in a ‘copy cat’ effect where hotels seek to have pet lemurs on the premises due to the perception that this will increase their income. It also requires an acknowledgment that some of these entities are trying to keep lemurs in good captive conditions (though the majority are not able to [22]), and that they could be used to facilitate conservation efforts under the right, albeit resource-intensive, circumstances (i.e. with continued assistance, monitoring, permitting, etc.).

In an ideal situation, increased and targeted enforcement mechanisms could be used to address highly-visible lemur ownership. Increased enforcement would result in the owners of visible pet lemurs facing public consequences for their illegal behavior; such a targeted program of enforcement would send a strong message about the illegality of pet lemur ownership. However, and similar to a national enforcement of lemur hunting bans [35], it is unlikely–for various reasons (e.g., lack of resources, lack of political will, other large-scale threats faced by lemurs)–that the enforcement of pet lemur ownership laws in Madagascar will improve. More broadly, there is a real need to build capacity and mobilize resources so that government agencies can address this issue, should there be the willpower to do so. It is currently unclear how legal captive facilities are permitted and how illegal owners can seek permits [19], in addition to a lack of oversight of permitted facilities and no apparent standard procedures for care of facilities that keep lemurs legally (ML pers. obsv.). It is also evident that there is a lack of capacity and resources at all levels of government to work on this issue, including outreach to owners and enforcement (up to confiscation). Following confiscation, there are a lack of sufficient facilities to house and rehabilitate lemurs and no adequate mechanism for using these in-country captive facilities to systematically support conservation efforts. Additionally, since most pet lemurs cannot survive in the wild, confiscated animals must often be cared for throughout the remainder of their lives, which for some species spans over 20 years (e.g. *L*. *catta*).

Above and beyond working with the Malagasy government, conservation programming could be used to target: 1) the general public; 2) tourists; and 3) the owners of pet lemurs. For the general public, outreach campaigns could be used to reinforce the message that lemurs should not be kept as pets. There are some examples of how this could be undertaken. In 2016, the authors of this paper organized the ‘Keeping Lemurs Wild’ campaign as a pilot project that was launched, nationwide, to reach the general public through posters, radio spots, and community presentations. The campaign placed 675 waterproof posters (in 8 languages and dialects) in 27 cities, towns, and villages in Madagascar and offered free, downloadable versions of the posters and other educational materials online. As a more recent example, in 2018, USAID headed the ‘Wildly Beautiful’ campaign, with contributions from several of the authors on this paper, which aimed to combat the illegal live trade of lemurs as pets in the country. For tourists, there could be targeted outreach about the illegal status of many captive lemurs in Madagascar. Tourists are unwittingly creating a demand for pet lemurs, even if they are unaware of the negative impacts that their actions are having on wildlife [36]. Tourists typically rely on information given to them by destination guides and assume that regulating bodies are overseeing wildlife interactions [36]. Therefore, outreach campaigns to tourists, travel agencies, and tour guides could be used to ensure that these individuals avoid providing incentives to individuals and/or entities to keep lemurs as pets for money-making purposes. Finally, outreach to the owners of pet lemurs in Madagascar (both legal and illegal) is a much-needed conservation action. With suggestions detailed in the literature [22], these kinds of targeted programming are increasingly necessary given the large number of lemurs that we estimate are kept as illegal pets.

It is also clear that conservation programming must consider which species are most affected by pet lemur ownership. Some species, like *L*. *catta*, appear to be disproportionately affected, both in terms of their popularity as pets and in terms of the impacts the illegal pet lemur trade could have on the species (on top of existing threats such as habitat degradation and hunting) [13,14]. There are probably other species–like the Critically Endangered *Hapalemur alaotrensis* (Alaotran gentle lemur)–where even just a small amount of localized live extraction for pet ownership would be enough to cause localized population extinctions over time. Unfortunately, we were not able to address threats to other species, including rare species, in this study due to limitations of the dataset. Regardless, it is important to consider species conservation status and estimated population size when deciding where to target outreach and conservation programming.

In summation, the illegal trade in pet lemurs in Madagascar significantly threatens several species. This is an added pressure to animals already facing rapid loss of habitat, and for some species or locations, unsustainable hunting for bushmeat [8]. Large and important areas for biodiversity conservation in Madagascar have experienced recent illegal clearings for agriculture [37] or mining purposes [38], and/or are devoid of lemurs because of illegal over-hunting and possibly extraction for the pet [12,14], despite the animals being present as recently as a decade ago. These trends of targeted biodiversity loss resulting from illegal activity needs to be urgently addressed by the government of Madagascar [39].

## Supporting information

S1 Table Town-level data (for towns with more than 5,000 people) on the number of pet lemurs owned and seen in the recent past. (XLSX)

S2 Table Genus/Species of pet lemurs reported by households during 2016 surveys. (DOCX)
