# Supplementary material for: A national survey of household pet lemur ownership in Madagascar
Source: PLoS One. 2019 May 8;14(5):e0216593. doi: 10.1371/journal.pone.0216593 (PMC6506143; doi:10.1371/journal.pone.0216593)
Supplement: S2 Table — (DOCX) [file pone.0216593.s002.docx]

**Table S2.** Genus/species of pet lemurs reported by households during 2016 surveys. Not all respondents were able to guess which genus/species they had seen (from a two-page series of photographs meant to broadly represent the different genera of lemur species).

| Town | *Allocebus* spp. | *Avahi* spp. | *Cheirogaleus* spp. | *Eulemur* spp. | *Hapalemur* spp. | *Indri indri* | *Lemur catta* | *Lepilemur* spp. | *Microcebus* spp. | *Mirza* spp. | *Prolemur simus* | *Propithecus* spp. | *Varecia* spp. |
| --- | --- | --- | --- | --- | --- | --- | --- | --- | --- | --- | --- | --- | --- |
| Ambositra |  | 1 | 3 | 14 | 2 |  | 9 | 3 |  |  | 2 | 1 | 1 |
| Anakao |  |  | 1 |  |  |  | 30 |  |  |  | 1 |  |  |
| Andasibe |  |  |  | 3 |  |  | 1 |  | 3 |  |  |  |  |
| Antananarivo |  |  |  | 2 | 1 |  | 3 |  |  | 1 |  | 2 |  |
| Antsirabe |  |  | 2 | 7 |  | 1 | 10 |  |  |  |  | 1 |  |
| Beforona |  |  |  | 3 |  |  | 2 |  |  |  |  |  | 1 |
| Efotse |  |  |  |  |  |  | 8 |  |  |  |  |  |  |
| Fianarantsoa |  |  |  | 7 | 4 |  | 33 |  | 1 | 1 | 2 | 1 | 1 |
| Tôlanaro (Fort Dauphin) |  |  |  | 13 |  |  | 18 |  |  |  |  |  |  |
| Moramanga |  | 1 | 1 | 3 | 5 | 1 | 1 |  | 1 | 1 | 3 | 1 |  |
| Toamasina (Tamatave) | 1 |  | 2 | 4 | 5 |  | 1 |  | 1 | 1 | 1 | 1 | 6 |
| Toliara (Tulear) |  |  |  |  |  |  | 16 |  |  |  |  |  |  |
| TOTAL | 1 | 2 | 9 | 56 | 17 | 2 | 132 | 3 | 6 | 4 | 9 | 7 | 9 |
